# Prediction of Disease Causing Non-Synonymous SNPs by the Artificial Neural Network Predictor NetDiseaseSNP

**DOI:** 10.1371/journal.pone.0068370

**Published:** 2013-07-25

**Authors:** Morten Bo Johansen, Jose M. G. Izarzugaza, Søren Brunak, Thomas Nordahl Petersen, Ramneek Gupta

**Affiliations:** 1 Center for Biological Sequence Analysis, Department of Systems Biology, Technical University of Denmark, Kongens Lyngby, Denmark; 2 Novo Nordisk Foundation Center for Biosustainability, Technical University of Denmark, Hørsholm, Denmark; 3 Novo Nordisk Foundation Center for Protein Research, University of Copenhagen, Copenhagen, Denmark; University of Utah, United States of America

## Abstract

We have developed a sequence conservation-based artificial neural network predictor called NetDiseaseSNP which classifies nsSNPs as disease-causing or neutral. Our method uses the excellent alignment generation algorithm of SIFT to identify related sequences and a combination of 31 features assessing sequence conservation and the predicted surface accessibility to produce a single score which can be used to rank nsSNPs based on their potential to cause disease. NetDiseaseSNP classifies successfully disease-causing and neutral mutations. In addition, we show that NetDiseaseSNP discriminates cancer driver and passenger mutations satisfactorily. Our method outperforms other state-of-the-art methods on several disease/neutral datasets as well as on cancer driver/passenger mutation datasets and can thus be used to pinpoint and prioritize plausible disease candidates among nsSNPs for further investigation. NetDiseaseSNP is publicly available as an online tool as well as a web service: http://www.cbs.dtu.dk/services/NetDiseaseSNP

## Introduction

A non-synonymous single nucleotide polymorphism (nsSNP) is a single nucleotide substitution occurring inside the coding region of a gene which causes an amino acid substitution in the corresponding protein product. In the current work, we include somatic single point mutations in this overall concept. An amino acid change can cause a structural or functional change in the protein product which potentially results in a minor or major phenotypic change. It is also entirely possible that a nsSNP has no phenotypic effect at all [1]. With the recent increase in the amount of nsSNP data [2]–[4], algorithms for the automatic prediction and prioritization of these phenotypic consequences are therefore valuable. For example, the Sorting Intolerant from Tolerant (SIFT) algorithm [5]–[8] is arguably the most well recognized tool for prediction of disease causing nsSNPs due to its high performance and its easy applicability to large datasets, which is a result of an excellent alignment generation step which is specialized for analysing disruption by nsSNPs of conserved sequences. Furthermore, SIFT constitutes the basis for other prediction methods where the output categories have further been expanded from classifying between disease causing and neutral to predict activity changes [9]. More recently, machine learning algorithms have found practical use for many tasks within sequence analysis and pattern recognition due to their ability to capture sequence correlations, which are present in numerous acceptor site motifs and other functional features [10]. Several methods for predicting phenotypic changes caused by nsSNP have been published during the last decade such as PolyPhen-2 [11], [12], SNAP [13] and Mutation Assessor [14]. Some methods are specifically tuned for certain diseases such as cancer [15] or particular protein subfamilies, as is the case of the methods to predict the pathogenicity of mutations in protein kinases [16], [17]. In addition, several meta-servers have been published lately. These methods combine a weighted selection individual classifiers, such as the ones presented above, and generate a consensus prediction of the impact of mutations, which usually yields increased performances. Condel [18], Consensus [19] and PON-P [20] constitute successful examples of the latter.

Another tool that maps functional changes caused by nsSNPs is EPipe. The EPipe server is a versatile tool which performs comparative analysis of protein variants resulting from genomic variation (e.g. nsSNPs), somatic mutations, alternative splicing or protein families from one or more organisms. The server is publicly available at http://www.cbs.dtu.dk/services/EPipe. The input proteins are first processed by a number of analysis and prediction methods and results are mapped onto a multiple alignment showing only the differential protein feature space of e.g. post-translational modification (PTM) sites or protein sorting signals.

Several public databases containing nsSNP data with phenotypic impact exist and among the most widely used are the Single Nucleotide Polymorphism database (dbSNP) [21] and the Universal Protein Resource (UniProt) [22]. Commercial databases such as the Human Gene Mutation Database (HGMD) Professional [23] provide valuable disease associated information. SNPedia (http://www.snpedia.com) is an example of a database that contains SNP data with linked phenotypes which are both disease and non-disease related. Furthermore databases of SNPs or mutations for specific diseases such as cancer also exist e.g. the Catalogue Of Somatic Mutations In Cancer (COSMIC) [24]. The COSMIC dataset can be assumed to be enriched for cancer driver mutations as compared to large scale somatic mutation discovery datasets which can be expected to contain a fair number of passenger mutations [9]. These databases can be used for training and evaluation of methods for prediction of phenotypic changes caused by nsSNPs that have a functional phenotypic impact and can be causative in diseases.

Among structural information related features protein surface accessibility of the SNP site obtained from 3D-structure has been shown to be important for prediction of disease causing nsSNPs [1], [11]–[13], [25]–[27]. When no 3D-structure is available, a prediction of surface accessibility may be expected to increase the performance of disease SNP predictions.

In the current study we present NetDiseaseSNP, a sequence conservation-based predictor of the pathogenicity of mutations which exploits the predictive power of artificial neural networks (ANNs). Our method derives sequence conservation from a PSSM based on the alignment algorithm of SIFT, which is complemented with the calculation of surface accessibility by our previously published predictor NetSurfP [28]. This approach provides NetDiseaseSNP with the potential to extract all relevant information directly from protein sequences. In addition, we show that our predictor outperforms some of the current state-of-the art disease SNP predictors in different scenarios.

## Results and Discussion

### Feature selection and description of the Neural Network

Here we present NetDiseaseSNP, a method for the prediction of the pathogenicity of mutations based on sequence conservation and surface accessibility. The system derives sequence conservation from a PSSM based on the alignment algorithm of SIFT. This information is complemented with the calculation of surface accessibility by our previously published predictor NetSurfP [28]. Finally, the system exploits the predictive power of artificial neural networks (ANNs) to calculate the likelihood of mutations to alter protein function.

Sequence conservation and surface accessibility have proven to be valuable for methods predicting phenotypic changes caused by nsSNPs [1]. Evolutionary information is typically gathered by an alignment step and used to produce a position specific scoring matrix (PSSM). For example, the alignment algorithm of SIFT is designed to gather an optimal set of distantly related sequences [7] and in doing so, it collects sequence specific information which can be used to distinguish conserved from variable positions in the protein investigated. In the absence of specific alignments for the protein being considered, generic substitution matrices such as Blosum62 may be employed, albeit with a lower performance [5].

We evaluated the performance of PSSMs generated by both SIFT and PSI-BLAST when used as input to the ANNs. We observed that the PSSMs generated by SIFT produced better results and consequently were preferred for the predictor. We evaluated the performance when NetSurfP uses its own PSSMs or the ones generated by SIFT PSSMs with a two-sided binomial test where the investigated SNPs were SNPs predicted in opposite categories when the NetSurfP output was either used as input or omitted. Polymorphisms predicted correctly when the NetSurfP output was used as input was thus counted as a success and polymorphisms predicted correctly when no NetSurfP output was used were counted as a failure. In both cases, we observed a significant increase in the performance. The p-values for NetSurfP PSSMs and SIFT PSSMs were found to be: 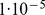 and 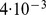, respectively and that the results were comparable (p-value 0.17).

Different input to the ANNs was tested and the optimal encoding of the features was found to be an extensive feature-space comprising 31 properties calculated directly from the protein sequences. Each of these properties corresponds to an input neuron in the ANN used to build NetDiseaseSNP as follows: Two input neurons receive the log-odds scores for the native and mutant amino acids, respectively. A log-odds score below zero suggests that the given amino acid is disfavoured at the given position.

Additionally, 20 input neurons which receive the log-odds scores for each amino acid in the analyzed position in the sequence alignment i.e. the row in the PSSM corresponding to the SNP position. This gives our predictor the potential to derive a measure of the sequence conservation at the SNP site and to learn which amino acids are similar or dissimilar meaning that these amino acids are favourable or disfavourable substitutions with respect to e.g. physiochemical and structural properties, from a purely data driven approach. The combination of these 22 log-odds scores thus makes it possible for our predictor to compare the native and SNP amino acids to the 20 standard amino acids based on the values of the log-odds scores and investigate which of them are more likely tolerated according to the alignment. Based on this NetDiseaseSNP has the potential to infer physiochemical and structural properties for the native and SNP amino acid.

The significance of the above mentioned aspects of the alignment will then be correlated with the disease causing potential of the SNP by our predictor. This data driven learning process can be assumed — provided enough data — to give a better and more detailed picture of these correlations than e.g. encoding the native and SNP amino acids in a reduced alphabet based on physiochemical properties where e.g. a combined log-odds score could be calculated for E and D.

Providing the 20 standard amino acid log-odds scores plus the log-odds scores for the native and SNP amino acids hence gives a more informative and detailed description of the environment for the site of the SNP than just using the normalized probability for the SNP amino acid for deciding whether a SNP is disease causing or not — as is done by the SIFT algorithm. The same arguments as mentioned above for physiochemical properties apply for e.g. structural properties (e.g. small/large amino acid) of native and SNP amino acids.

NetDiseaseSNP encodes in one input neuron the SIFT score (normalized probability) for the SNP amino acid. This score is sufficient on its own to discriminate between neutral and disease variants very accurately [5]. Another input neuron encodes the median conservation score (RcMedian) from the SIFT output. This feature is discussed in the original publications [6], [7]. It is worth pointing out that this score can be regarded as a confidence score for the SIFT score. The optimal diversity of the sequences in the alignment is achieved for a value of the RcMedian score between 2.75 and 3.00. For values above 3.00 the diversity in the alignment becomes too small and the performance of the predictions made by SIFT based on the SIFT score decreases. On the other hand if the RcMedian score is below 2.75 then the alignment has become too diverse and SIFT will in these cases predict too many neutral SNPs and hence predict more experimentally verified disease SNPs as neutral SNPs. NetDiseaseSNP is thus trained to have the potential to make its own conclusions about how significant the SIFT score value is and thus to decide if it is better to trust e.g. the surface accessibility prediction more in cases where the RcMedian score indicates that the SIFT score is less reliable. NetDiseaseSNP is also provided (one input neuron) with the conservation score for the SNP site which gives a measure of how conserved all amino acids are at the SNP site as opposed to the SIFT score which only compares the probability for the SNP amino acid and the most frequent amino acid in the alignment. The SIFT and conservation scores may thus supplement each other by highlighting slightly different aspects of the conservation at the SNP site in the alignment. The conservation score for the SNP site is thus another example of how attempts are made for our predictor to use all relevant information which is available in the alignment created by SIFT.

Finally, NetSurfP predictions for the non-mutated native query sequences are also encoded. Two neurons receive the relative surface accessibility and the associated reliability score of the mutated residue. Two additional pairs of input neurons process this information for the residues directly adjacent to the mutated one. The main advantage of using predicted surface accessibility as opposed to e.g. data from a PDB file is the possibility to generate predictions for proteins where no 3D structure is yet available, which is often the case. Consequently, a total of 6 input neurons encode the information from NetSurfP.

The other input features may as discussed above provide information about the physiochemical and structural significance of the amino acid change which can then be correlated with the surface accessibility of the SNP site. This type of correlations would provide information about e.g. a hydrophobic amino acid inside the protein being changed to a charged amino acid — such substitutions could potentially be damaging to the protein.

### Ranking mutations with NetDiseaseSNP

**Figure 1:**
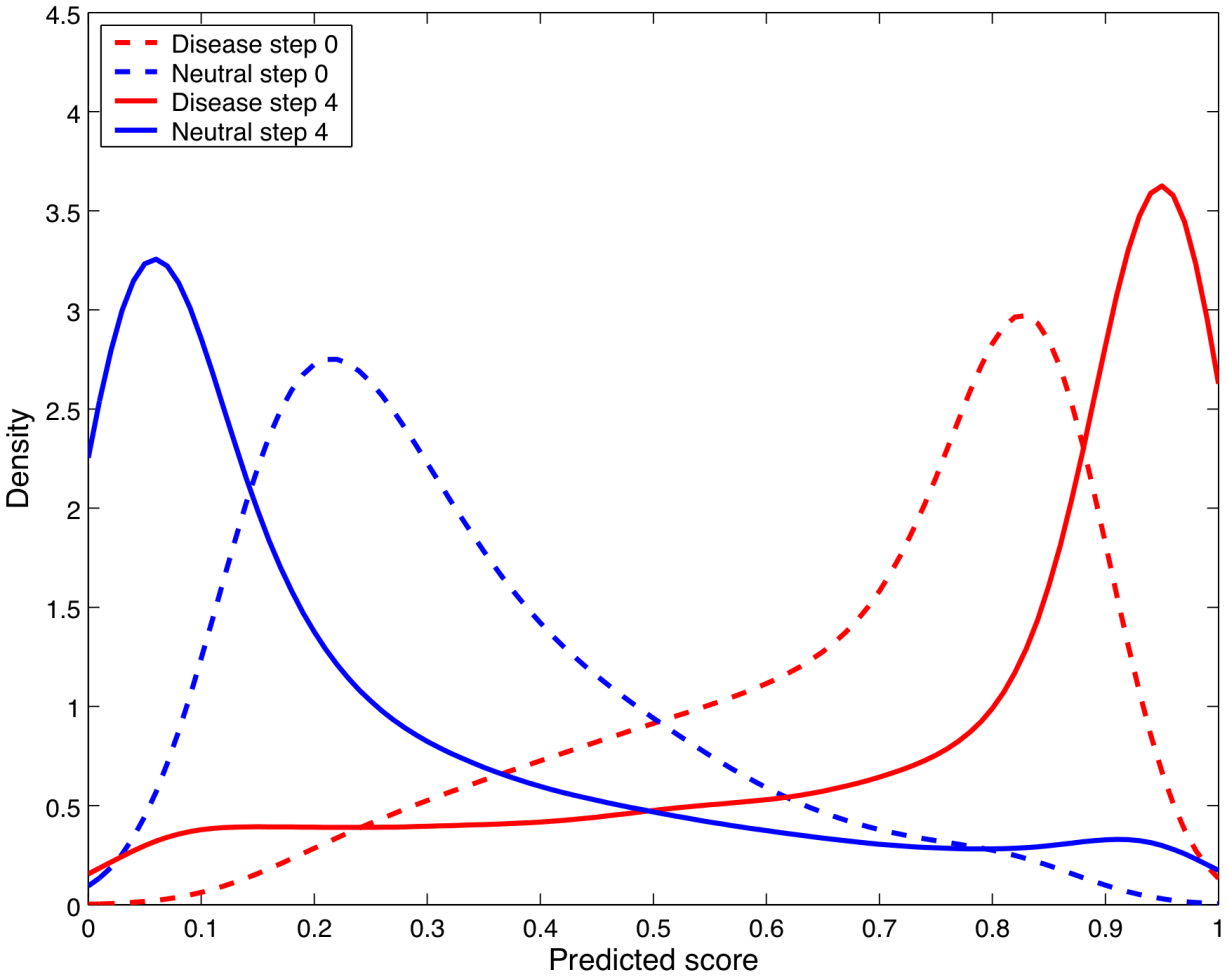
Softening of target values procedure. Density plot showing the change in the distribution of the output values for known disease and neutral SNPs in the running evaluation set during the softening of target values procedure. Step 0 is the distribution of output values before the procedure and step 4 is the distribution at the end of the procedure for the final version of NetDiseaseSNP. Predicted scores above the threshold of 0.5 are true positives for data shown in red graphs, whereas scores above 0.5 in the blue graphs represent false positives. It is seen that predicted scores are dragged more to the extreme ends (0–1) after applying the procedure ‘softening of target values’.

The output score of NetDiseaseSNP can be used to rank and prioritize polymorphisms for further investigation. The ANNs of our predictor will generate an output value close to 1 if the combination of features describing that particular mutation suggests that it might be involved in disease, and close to 0 for neutral mutations. The distance to the prediction threshold (0.5) can be used as an indication of the reliability of the prediction, as observed in Figure 1.

### Softening of target values

The target values in the training set were optimized by a procedure that we refer to as softening of target values. This optimization resulted in a better separation of predicted scores (Figure 1). For each step in the softening procedure an increase in performance was observed and it was checked that this increase in performance was significant by performing a two-sided binomial test (data not shown). In this test a correct prediction for the given SNP in the current step and a wrong prediction in the previous step were counted as a success, and a wrong prediction for the given SNP in the current step and a correct prediction in the previous step were counted as a failure. It should be noted that the softening procedure was only applied to the training set, and not test or evaluation sets. 36 ANNs were selected from the last step of the softening procedure and used to build the final version of NetDiseaseSNP. The number of hidden neurons for these ANNs lies in the range from 8 to 38. This procedure is explained in detail in the Materials and Methods section.

The softening of target values procedure is used to move the target values in the training set for the worst wrongly predicted SNPs closer to the threshold value of 0.5 to reduce the contribution of these to the error function in the backpropagation algorithm. The new target values are generated from the average test output values — and not from the average evaluation output values to avoid optimization on the evaluation set. The idea behind the softening of target values procedure is thus to correct for polymorphisms in the dataset which are wrongly classified (disease/neutral) in the experimental studies e.g. undiscovered disease polymorphisms but also polymorphisms which are not directly causative of a disease e.g. SNPs dependent on other SNPs to cause a disease. The increased density in Figure 1 of FNs with output values close to 0 and FPs with output values close to 1 when comparing step 0 and 4 in the softening of target values procedure could be attributed to such SNPs.

By softening the targets values stepwise starting with the most incorrectly predicted polymorphisms the ANNs will gradually become more robust in their predictions thus making it possible to address less wrongly predicted polymorphisms. The increased robustness of the ANNs is evident from the significant increase in performance for each step in the procedure and the better separation of the output values as shown in Figure 1.

### Performance of NetDiseaseSNP

We evaluated the performance of NetDiseaseSNP using a 4-fold cross-validation approach on a dataset comprising 58872 mutations from HGMD and UniProt for which their character as disease-causing or neutral has been described. This dataset was complemented with 18468 neutral human-rodent differences to balance the resulting datasets (Table 1). Further details on the generation of the datasets and the construction of the associated PSSMs can be found in the Materials and Methods section. We evaluated the performance achieved by our classifier according to well established measures [29] and we observed that NetDiseaseSNP is capable of classifying disease-causing mutations from neutral polymorphisms satisfactorily. Accuracy (0.82), precision (0.83), sensitivity (0.80) and specificity (0.83) are fairly high and well balanced. MCC and F-score for this dataset were 0.64 and 0.81 respectively. These results have been summarized in Table 2.

**Table 1:** Composition of the training sets.

	Neutral		Disease	
*Source*	*SNPs*	*Proteins*	*SNPs*	*Proteins*
UniProt	20202	7513	6904	1847
HGMD	—	—	31766	1593
Human-rodent	18468	2260	—	—
All	38670	7979	38670	3440

Contribution of each source database to the training datasets. The table shows the number of SNPs, as well of the number of affected proteins, in each of the prediction categories, namely, neutral and disease-associated.

**Table 2:** Benchmark of NetDiseaseSNP.

*Method*	*N*	*Accuracy*	*Precision*	*Sensitivity*	*Specificity*	*F-score*	*MCC*
NetDiseaseSNP	77340	0.82	0.83	0.80	0.83	0.82	0.64
SIFTnd	75647	0.82	0.83	0.79	0.84	0.81	0.63
SIFTsd	24584	0.67	0.41	0.54	0.72	0.47	0.24
SNAP	25141	0.51	0.33	0.84	0.40	0.48	0.22
Polyphen2	11012	0.61	0.09	0.81	0.60	0.17	0.18
MutationAssessor	40693	0.64	0.30	0.86	0.60	0.44	0.34

Performance of NetDiseaseSNP and other state-of-the art predictors. The evaluation was performed on all variants in the evaluation set. This includes data obtained from Blosum62 matrices.

In addition, we evaluated the performance of our classifier only in the cases where there is abundant evolutionary information so that SIFT PSSM can be generated. For this subset of mutations the performance measures increase substantially. Specificity and Sensitivity become 82% and 85% respectively, yielding a F-score of 0.83, whereas MCC rises to 0.67 as described in Table 3. Furthermore, the performance of the classifier can go to MCC values reaching 0.70 when a consensus prediction between SIFT and NetDiseaseSNP can be obtained, which is by far the most common scenario. Contrarily, when a SIFT alignment has been used as input to NetDiseaseSNP, but the two predictors disagree on the given prediction the performance of NetDiseaseSNP is MCC = 0.25. Our predictor has some prediction potential for SNPs generated from a Blosum62 matrix, however, the performance dropped significantly (MCC = 0.42) and predictions should be used with some caution. Since the performance of NetDiseaseSNP is low on these two latter types of SNPs, they are therefore not included in the default output from NetDiseaseSNP.

**Table 3:** Benchmark of NetDiseaseSNP: SIFT PSSMs.

*Method*	*N*	*Accuracy*	*Precision*	*Sensitivity*	*Specificity*	*F-score*	*MCC*
NetDiseaseSNP	67119	0.83	0.84	0.82	0.85	0.83	0.67
SIFTnd	67119	0.82	0.83	0.79	0.84	0.81	0.63
SIFTsd	22020	0.68	0.41	0.54	0.73	0.46	0.25
SNAP	22417	0.52	0.32	0.83	0.41	0.46	0.22
Polyphen-2	10042	0.61	0.07	0.80	0.60	0.14	0.16
MutationAssessor	35657	0.64	0.29	0.86	0.60	0.43	0.33

Performance of NetDiseaseSNP and other state-of-the art predictors. The evaluation was performed only on the variants for which a SIFT PSSM was available. This excludes data obtained from Blosum62 matrices.

Additionally, we benchmarked NetDiseaseSNP against a number of state-of-the-art predictors of the pathogenicity of mutations including SIFT [5], PolyPhen-2 [11], [12], SNAP [13] and Mutation Assessor [14]. These methods were chosen because they are among the most widely used methodologies but also because they approach the pathogenicity prediction problem from different angles as we have described previously. The results of this analysis can be found in Table 2 and Table 3 depending on whether Blosum62 generated predictions are considered or excluded from the analysis, respectively. Interestingly, NetDiseaseSNP outperformed the aforementioned predictors and demonstrated a fair balance between specificity and sensitivity.

For the rarely occurring sequences longer than 2000 amino acids multiple alignments are harder to obtain through SIFT and Blosum62 matrix data is always used by NetDiseaseSNP to encode variations in such proteins. Splitting such long proteins into their functional domains will allow SIFT to generate the required PSSMs.

A detailed performance comparison between NetDiseaseSNP and SIFT is available in Table S1 in File S1 .

### Prediction of cancer driver and passenger mutations

In order to test the ability of distinguishing driver from passenger mutations we evaluated NetDiseaseSNP with the same dataset used for the development of CanPredict [15], a cancer-associated missense mutation predictor.This dataset consists of cancer mutations from the COSMIC cancer dataset which are assumed to be driver mutations and SNPs from dbSNP with a 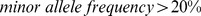 which are considered passenger mutations. After verifying the nsSNPs from dbSNP against Ensembl v.54, NCBI Build 36 our final dataset consisted of 997 driver mutations and 3404 passenger mutations.

From the results described in Table 4 we can conclude that NetDiseaseSNP can distinguish cancer driver mutations from passenger mutations very accurately (0.85). This observation also stands true when the ability to identify drivers is evaluated: precision (0.81) and recall (0.62). This well balanced performance leads to a f-score of 0.70 and a MCC of 0.61. We benchmarked the prediction capabilities of NetDiseaseSNP with respect to other state-of-the-art methodologies to predict the pathogenicity of mutations. These included SIFT [5], PolyPhen-2 [11], [12], SNAP [13] and Mutation Assessor [14]. From the analysis of the results, we can conclude that NetDiseaseSNP outperformed the rest of methods. In particular, we observed that the predictions are balanced in terms of specificity and sensitivity, whereas the other predictors either show a tendency to predict a big number of mutations as disease causing, i.e. allowing for erroneous predictions, or to be very conservative with their predictions, i.e. predict fewer mutations for which their pathogenicity is very clear. The performance of NetDiseaseSNP on mutations where Blosum62 matrix data has been used as input drops significantly (MCC = 0.11). We can conclude from this result that the predictions generated by NetDiseaseSNP from Blosum62 matrix data should be used with caution. A detailed performance comparison between NetDiseaseSNP and SIFT can be found in Table S3 in File S1.

**Table 4:** Benchmark of NetDiseaseSNP: Cancer drivers and passengers.

*Method*	*N*	*Accuracy*	*Precision*	*Sensitivity*	*Specificity*	*F-score*	*MCC*
NetDiseaseSNP	4401	0.85	0.62	0.81	0.86	0.70	0.61
SIFTnd	4036	0.84	0.63	0.79	0.85	0.70	0.60
SIFTsd	2778	0.78	0.37	0.64	0.81	0.47	0.36
SNAP	2835	0.57	0.24	0.85	0.51	0.37	0.26
Polyphen-2	1686	0.78	0.06	0.85	0.78	0.11	0.19
MutationAssessor	1587	0.66	0.19	0.86	0.64	0.31	0.29

Performance of NetDiseaseSNP and other state-of-the art predictors on the cancer-specific dataset from CanPredict [15].

**Figure 2:**
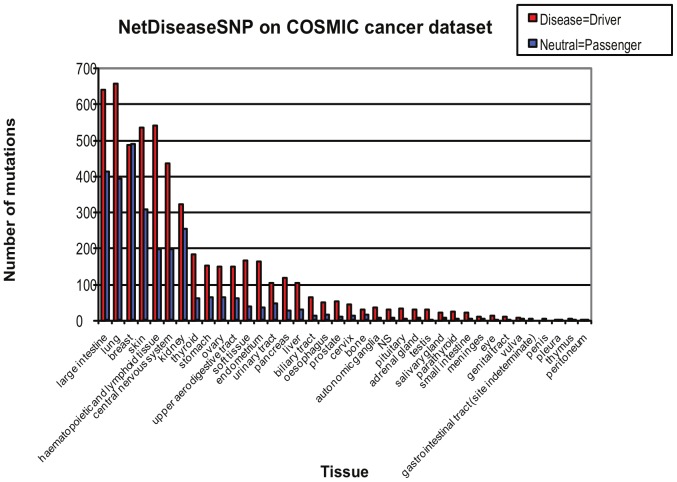
Prediction by NetDiseaseSNP on COSMIC. Number of predicted passenger (neutral) and driver (disease) mutations for the different tissue types in the COSMIC cancer dataset. Our recommendation that predicted disease mutations are ‘drivers’ further suggests that while breast cancer shows almost an equal number of driver and passenger mutations other cancer types are more enriched for ‘drivers’ — at least in the COSMIC dataset.

The ability of our predictor to predict cancer driver mutations from passenger mutations was initially tested on the COSMIC cancer mutation dataset [24] where we in the test assumed that variants predicted to be disease mutations are predicted driver mutations and that variants predicted to be neutral mutations are predicted passenger mutations. The COSMIC dataset can be assumed to be enriched for driver mutations as compared to large scale somatic mutation discovery datasets which can be expected to contain a fair number of passenger mutations [9]. NetDiseaseSNP was hence shown to predict significantly more driver mutations than passenger mutations in the COSMIC dataset. Notice though that some predicted disease mutation might not be driving the cancer development but instead cause other diseases. Figure 2 shows the number of predicted passenger (neutral) and driver (disease) mutations for the different tissue types in the COSMIC cancer dataset. Interestingly, the only tissue type which has approximately the same number of predicted passenger and driver mutations is breast tissue while the other tissue types have more predicted driver mutations than passenger mutations. The results are discussed in detail in Table S2 in File S1.

### Implementation of the method as a web server

NetDiseaseSNP is publicly available as an online tool as well as a web service: http://www.cbs.dtu.dk/services/NetDiseaseSNP. Sequences are submitted in fasta format, whereas variant data is encoded according to the following space-separated format: ‘Accession’, ‘native amino acid’, ‘position’, ‘variant amino acid’. Individual queries can be submitted by pasting sequence and variation data in the corresponding text-boxes, however, it is also possible to perform batch submissions where a fasta file and variant file can be uploaded from the local computer. The jobs are parallelized and handled by a queuing system. Performing the whole calculations on a standard protein of 250 amino acids with any number of variants is expected to provide results within 15 minutes in the currently available hardware. In addition, a cache has been implemented to speed up the calculation of recurrent queries. The output from NetDiseaseSNP contains the original input as well as the predictions in a simple space-separated format. The predictions consist of the NetDiseaseSNP score and the predicted category for each mutation (disease/neutral). NetDiseaseSNP score ranges from 0 to 1 where scores 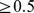 are indicative of the mutation being involved in disease, whereas neutral mutations are associated to values below this threshold. The default output from NetDiseaseSNP only displays predictions where NetDiseaseSNP and SIFT agree. This default behaviour includes most variants and allows the user to focus only on the most reliable predictions since NetDiseaseSNP has the highest performance for such variants. Nevertheless, this default settings can be easily customizable to include variants where NetDiseaseSNP and SIFT disagree or predictions based on Blosum62 matrices.

### Conclusion

The amount of variant data is growing fast and algorithms for predicting and prioritizing phenotypic changes caused by these variants are therefore becoming increasingly valuable. We have developed NetDiseaseSNP, a sequence conservation-based prediction of the pathogenicity of mutations that exploits the predictive power of artificial neural networks (ANNs). NetDiseaseSNP derives sequence conservation from a PSSM based on the alignment algorithm of SIFT, which is complemented with the calculation of surface accessibility by NetSurfP [28]. This combined set of features describes the mutations very efficiently and allows our method to predict the implication of mutations in disease very accurately. Additionally, we have shown that NetDiseaseSNP is able to discriminate between cancer driver and passenger mutations accurately. In addition, we have show that our predictor outperforms some of the current state-of-the art disease SNP predictors in both datasets.

In summary, we have thus demonstrated that our predictor can be used to pinpoint and prioritize plausible disease candidates among nsSNPs for further investigation. NetDiseaseSNP is publicly available at http://www.cbs.dtu.dk/services/NetDiseaseSNP

## Materials and Methods

### Generation of the training dataset

The training dataset was obtained from HGMD Professional [23] and UniProt [22]. In order to increase the quality of the training datasets, we performed some filtering and curation on these datasets. nsSNPs from HGMD which were reported as not necessarily causative of disease were discarded. These were identified by a matching ‘assoc’ or ‘?’ in the disease field. After this initial filtering, we considered 32484 disease mutations in 1606 proteins from HGMD and 18884 disease mutations and 21851 neutral polymorphisms affecting a total of 8705 proteins from UniProt. A homology reduction procedure was applied to eliminate duplicated variations after the combination of the two different datasets. Iterative BLAST i.e. PSI-BLAST [30] was run for each protein in the HGMD and UniProt datasets with an 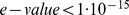, 3 iterations and the NCBI non-redundant protein database (nr, July 2008) was used as the sequence database. At this point and due to technical issues, two proteins longer than 9999 amino acids were excluded from the analysis and 78 disease and 214 neutral variations were not considered. The PSSMs from this PSI-BLAST run were used to combine the HGMD and UniProt datasets in a homology reduction procedure where the unique key for a SNP was defined as the 20 standard amino acid log-odds scores (rounded to nearest integer) in the PSSM for the SNP position and the one letter amino acid symbols for the native and SNP amino acids. Thus, a vector of length 22 was constructed for each SNP. Incongruences between the databases were resolved assuming that HGMD annotation is more accurate than UniProt, and that mutations annotated as disease and neutral might be undiscovered disease SNPs at the time of the analysis. 557 mutations from HGMD corresponded to neutral variations in UniProt and 24 variations were annotated both as neutral and disease-associated in UniProt. All these cases were considered disease-associated mutations in our training set. After the homology reduction step, 11682 UniProt disease SNPs were not considered due to similarity to an existing HGMD disease SNPs.

Even though ANNs correct for biases during training, experience has shown that better performance is achieved with balanced datasets comprising the same number of disease and neutral SNPs. Hence, we complemented our training set with neutral SNPs identified as mismatches in a pairwise alignment between human and rodent sequences. These pairwise alignments were generated from a BLAST search for each human UniProt proteins in the set, against the rodent (mouse and rat) sequences in UniProt. Then a reciprocal BLAST search was performed with the UniProt rodent sequence hits as query sequences against the human UniProt sequences. Both BLAST searches were constrained using 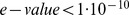 and 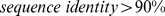. Mismatches from these alignments were assigned as additional neutral SNPs, provided that no known disease associated SNPs exist for the position of the given variation. From that set of human-rodent SNPs we randomly selected 18468 additional unique data points according to the homology criteria mentioned above until the two datasets presented the same number of mutations.

Table 1 shows the composition of the final training set which in total consists of 77340 SNPs in 10003 proteins. We have included this training set as part of the documentation of NetDiseaseSNP, from where it can be downloaded for further analysis.

### Generation of the cancer datasets

6724 non-synonymous cancer mutations were extracted from version v44 of the COSMIC dataset [24]. A dataset developed for the creation of the cancer-associated missense mutation predictor CanPredict [15] was downloaded from http://share.gene.com/mutation_classification/. The CanPredict dataset consists of cancer mutations from the COSMIC cancer dataset [31] which are assumed to be driver mutations and SNPs from dbSNP [21] with a minor allele 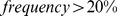 assumed to mimic passenger mutations. This dataset is publicly available in our web server.

### Generation of SIFT PSSMs

PSSMs were generated based on the previously mentioned PSI-BLAST run and for comparison PSSMs were also generated based on the alignment produced by SIFT using nr for the retrieval of related sequences and a median conservation score of 2.75. The SIFT PSSM was calculated by generating SIFT scores for all 20 standard amino acid symbols for all positions in the given protein:
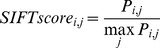
where 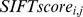 and 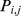 are the SIFT score and frequency, respectively, for the j'th standard amino acid symbol at position ‘i’ in the protein. Next scores were converted into probabilities for each position by dividing the score for the j'th standard amino acid symbol at position ‘i’ in the protein by the sum of all scores for the given i'th position:
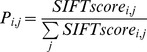



The probabilities were finally converted to log-odds scores by use of Blosum62 background frequencies. If a probability was zero then the minimum Blosum62 matrix log-odds score of −4 was used.

### Description of the classification features

The optimal input to the ANNs based on the data in the PSSM for the position of the SNP site was found to be:

- The 20 standard amino acid log-odds scores.
- The native amino acid log-odds score.
- The mutant amino acid log-odds score.
- The SIFT score for the mutation.
- The conservation score (Rc) for the given position.
- The median conservation score (RcMedian) calculated for the whole protein.
- The relative surface accessibility (RSA) and the associated reliability score for the mutated position and its two neighbouring positions as calculated in NetSurfP [28].

This gives an extensive input feature space containing 31 input features in each of the ANNs used to build NetDiseaseSNP. During testing of different input features the 20 standard amino acid log-odds scores and the log-odds scores for the SNP and native amino acids were the basic input features to which the other input features were added iteratively.

The log-odds scores for the 20 standard amino acid symbols and the native and SNP amino acids were taken from the PSSMs generated from the alignment produced by SIFT. However, when no SIFT prediction was available for the given SNP either because the protein is longer than 2000 amino acids or SIFT was not able to generate an alignment then Blosum62 matrix values were used for the log-odds scores and also for probabilities in the input to the ANNs.

The SIFT score for the SNP amino acid was calculated as the frequency of the SNP amino acid divided by the frequency of the most frequently occurring amino acid at the SNP position in the alignment - the SIFT score is thus a normalized probability.

A measure of the conservation at position ‘i’ in an alignment is given by:
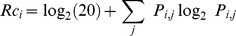
where 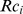 is the conservation score at position ‘i’ and 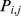 is the frequency at position ‘i’ for standard amino j in the PSSM for the protein. The conservation score is calculated for every position in the protein and the median value is used as input to the ANNs. Furthermore, the conservation score for the SNP position is also used as input to the ANNs.

The PSSM generated from the SIFT alignment is used by NetSurfP [28] to predict the surface accessibility for the native query protein without the SNP changes. The RSA-value and reliability score for RSA-value predicted by NetSurfP for the SNP position and the context in position -1 and +1 with respect to the SNP site is then used as input to the ANNs.

### Training of the classifier and evaluation of its performance

A standard feed forward architecture was used for the ANNs [10]. The ANNs thus comprised an input layer connected to a hidden layer and finally a single output neuron producing the output score. The back-propagation algorithm [32] was used for training the ANNs.

Cross-validation and a running evaluation were used to estimate the performance of the ANNs. In the cross-validation procedure, the dataset is divided into 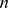 parts. One part is then selected for testing of the performance of the ANN during training, one part is selected for running evaluation, whereas the other 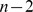 parts are used for training the ANN. This procedure is repeated 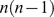 times, thus using each of the 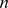 parts as a test set and running evaluation set. Consequently, the training dataset was split into four subsets for cross-validation. All SNPs from the same protein were placed in the same subset. This avoids overtraining of the ANNs due to non-biologically biases in the dataset related to specific proteins — such as e.g. protein length related features. Furthermore in order to balance the dataset proteins were partitioned in such a way that the subsets would approximately contain the same total number of SNPs and the same number of neutral and disease SNPs.

In order to provide an evaluation of the performance, we used 6 different measures commonly used for this purpose. They were reviewed in detail in a recent publication [29] and are described as follows:.
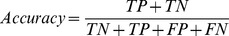


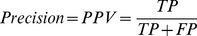


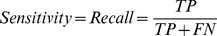


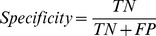


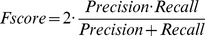


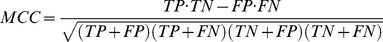
where TP is the number of correctly predicted disease SNPs (true positives), TN the number of correctly predicted neutral SNPs (true negatives), FP the number of SNPs falsely predicted to be disease SNPs (false positives), and FN the number of SNPs falsely predicted to be neutral SNPs (false negatives). The final MCC is calculated from the independent evaluation subsets only.

The best ANN architecture and best performing input features were selected by optimizing MCC based on sequences in the test sets.

### Softening of target values

When the optimal input to the ANNs had been found, the target values in the training set were optimized by a procedure we call softening of target values which was developed during this study.

In this procedure the target values of the most incorrectly predicted SNPs in the test, is modified with a new target value which is closer to the threshold value of 0.5 thereby reducing the contribution of these SNPs to the error function in the backpropagation algorithm. The new target values for these SNPs were calculated by the empirical formulas:
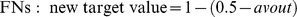


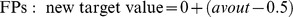
where avout is the average output value calculated for the test set in the cross-validation procedure. The most wrongly predicted SNPs were found by first selecting a cut-off on test average output values corresponding to a sensitivity and specificity of 95%. ANNs were then trained again as described above. The sensitivity and specificity cut-offs were then lowered in steps of 5% and new ANNs trained after each step where the target values in the training set were ‘softened’ by the two formula mentioned above based on the test set average output values from the previous step in the procedure. When the sensitivity and specificity cut-offs reached a value of 80% some of the new target values calculated by the formulas above started to change category (disease/neutral). The new target values were then recalculated for steps of 1% from the start value of 85% for the specificity and sensitivity cut-offs, however ANNs were not retrained after these steps. No changes in category were found for any SNPs for a sensitivity and specificity cut-off of 83% — so a sensitivity and specificity of 83% was used as the last step in the softening of target values procedure. For this last step the number of hidden neurons was varied between: 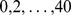 thus increasing the maximal number of hidden neurons during training allowing for more complex learning after the training set had been cleaned up. Furthermore, the three highest ranking sets of three ANNs with the same evaluation set were selected resulting in 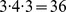 ANNs instead of 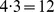 ANNs since averaging output from more well performing ANNs will generally give a higher performance.

## Supporting Information

File S1 **This file contains three supporting tables.** **Table S1aa** Performance of NetDiseaseSNP. (1) All SNPs where NetDiseaseSNP and SIFT agree on the prediction; (2) All SNPs where NetDiseaseSNP and SIFT disagree on the prediction; (3) All SNPs where SIFT is not able to generate a prediction; (4) All SNPs. **Table S1ab** Performance of SIFT. (1) All SNPs where NetDiseaseSNP and SIFT agree on the prediction; (2) All SNPs where NetDiseaseSNP and SIFT disagree on the prediction; (3) All SNPs where SIFT can generate a prediction. **Table S1ba** Performance of NetDiseaseSNP. (1) All SIFT data encoded SNPs where NetDiseaseSNP and SIFT agree on the prediction; (2) All SIFT data encoded SNPs where NetDiseaseSNP and SIFT disagree on the prediction; (3) All SIFT data encoded SNPs. **Table S1bb** Performance of SIFT. (1) All SIFT data encoded SNPs where NetDiseaseSNP and SIFT agree on the prediction; (2) All SIFT data encoded SNPs where NetDiseaseSNP and SIFT disagree on the prediction; (3) All SIFT data encoded SNPs where SIFT can generate a prediction. **Table S1ca** Performance of NetDiseaseSNP. (1) All Blosum62 data encoded SNPs where NetDiseaseSNP and SIFT agree on the prediction; (2) All Blosum62 data encoded SNPs where NetDiseaseSNP and SIFT disagree on the prediction; (3) All Blosum62 data encoded SNPs where SIFT is not able to generate a prediction; (4) All Blosum62 data encoded SNPs. **Table S1cb** Performance of SIFT. (1) All Blosum62 data encoded SNPs where NetDiseaseSNP and SIFT agree on the prediction; (2) All Blosum62 data encoded SNPs where NetDiseaseSNP and SIFT disagree on the prediction; (3) All Blosum62 data encoded SNPs where SIFT can generate a prediction. **Table S1da** Performance of NetDiseaseSNP. (1) All Blosum62 data encoded SNPs where SIFT predictions exist and NetDiseaseSNP and SIFT agree on the prediction; (2) All Blosum62 data encoded SNPs where SIFT predictions exist and NetDiseaseSNP and SIFT disagree on the prediction; (3) All Blosum62 data encoded SNPs where SIFT predictions exist. **Table S1db** Performance of SIFT. (1) All Blosum62 data encoded SNPs where SIFT predictions exist and NetDiseaseSNP and SIFT agree on the prediction; (2) All Blosum62 data encoded SNPs where SIFT predictions exist and NetDiseaseSNP and SIFT disagree on the prediction; (3) All Blosum62 data encoded SNPs where SIFT predictions exist and SIFT can generate a prediction. **Table S1e** Number of neutral and disease SNPs for each of different types of encoding for SNPs. The columns in the table are: Column 1: Input data to NetDiseaseSNP; Column 2: Protein is longer than 2000 amino acids; Column 3: SIFT data exists for the all SNPs; Column 4: Number of neutral SNPs; Column 5: Number of disease SNPs. The rows in the table are: (1) All SNPs both SIFT and Blosum62 data encoded SNPs; (2) SIFT data encoded SNPs; (3) Blosum62 data encoded SNPs; (4) Blosum62 data encoded SNPs where SIFT output data exists i.e. protein is longer than 2000 amino acids; (5) Blosum62 data encoded SNPs where SIFT output data does not exist. **Table S2a** Predictions by NetDiseaseSNP and SIFT. The rows in the table are: (1) Both NetDiseaseSNP and SIFT predict disease; (2) Both NetDiseaseSNP and SIFT predict neutral; (3) NetDiseaseSNP predicts disease and SIFT predicts neutral; (4) NetDiseaseSNP predicts neutral and SIFT predicts disease; (5) NetDiseaseSNP predicts disease and SIFT no prediction; (6) NetDiseaseSNP predicts neutral and SIFT no prediction; (7) Total number of mutations. **Table S2b** Predictions by NetDiseaseSNP on mutations where NetDiseaseSNP predicts the mutation to be a disease mutation and mutations are encoded with Blosum62 matrix data. The columns in the table are: Column 1: Description of the data in the row; Column 2: Protein is longer than 2000 amino acids; Column 3: All mutations at all positions in the protein are encoded with Blosum62 matrix data. The rows in the table are: (1) Protein longer than 2000 amino acids; (2) SIFT is not able to generate output for any mutation in this protein and the protein is shorter than 2000 amino acids; (3) SIFT is able to generate output for some mutations in this protein and the protein is shorter than 2000 amino acids; (4) Total number of mutations. **Table S2c** Predictions by NetDiseaseSNP on mutations where NetDiseaseSNP predicts the mutation to be a neutral mutation and mutations are encoded with Blosum62 matrix data. The columns in the table are: Column 1: Description of the data in the row; Column 2: Protein is longer than 2000 amino acids; Column 3: All mutations at all positions in the protein are encoded with Blosum62 matrix data. The rows in the table are: (1) Protein longer than 2000 amino acids; (2) SIFT is not able to generate output for any mutation in this protein and the protein is shorter than 2000 amino acids; (3) SIFT is able to generate output for some mutations in this protein and the protein is shorter than 2000 amino acids; (4) Total number of mutations. **Table S3a** Performance of NetDiseaseSNP. (1) All mutations where NetDiseaseSNP and SIFT agree on the prediction; (2) All mutations where NetDiseaseSNP and SIFT disagree on the prediction; (3) All mutations where SIFT is not able to generate a prediction; (4) All mutations. **Table S3b** Performance of SIFT. (1) All mutations where NetDiseaseSNP and SIFT agree on the prediction; (2) All mutations where NetDiseaseSNP and SIFT disagree on the prediction; (3) All mutations where SIFT can generate a prediction.
(DOC)
