# Supplementary material for: Prediction of Disease Causing Non-Synonymous SNPs by the Artificial Neural Network Predictor NetDiseaseSNP
Source: PLoS One. 2013 Jul 25;8(7):e68370. doi: 10.1371/journal.pone.0068370 (PMC3723835; doi:10.1371/journal.pone.0068370)
Supplement: File S1 — This file contains three supporting tables. Table S1aa Performance of NetDiseaseSNP. (1) All SNPs where NetDiseaseSNP and SIFT agree on the prediction; (2) All SNPs where NetDiseaseSNP and SIFT disagree on the prediction; (3) All SNPs where SIFT is not able to generate a prediction; (4) All SNPs. Table S1ab Performance of SIFT. (1) All SNPs where NetDiseaseSNP and SIFT agree on the prediction; (2) All SNPs where NetDiseaseSNP and SIFT disagree on the prediction; (3) All SNPs where SIFT can generate a prediction. Table S1ba Performance of NetDiseaseSNP. (1) All SIFT data encoded SNPs where NetDiseaseSNP and SIFT agree on the prediction; (2) All SIFT data encoded SNPs where NetDiseaseSNP and SIFT disagree on the prediction; (3) All SIFT data encoded SNPs. Table S1bb Performance of SIFT. (1) All SIFT data encoded SNPs where NetDiseaseSNP and SIFT agree on the prediction; (2) All SIFT data encoded SNPs where NetDiseaseSNP and SIFT disagree on the prediction; (3) All SIFT data encoded SNPs where SIFT can generate a prediction. Table S1ca Performance of NetDiseaseSNP. (1) All Blosum62 data encoded SNPs where NetDiseaseSNP and SIFT agree on the prediction; (2) All Blosum62 data encoded SNPs where NetDiseaseSNP and SIFT disagree on the prediction; (3) All Blosum62 data encoded SNPs where SIFT is not able to generate a prediction; (4) All Blosum62 data encoded SNPs. Table S1cb Performance of SIFT. (1) All Blosum62 data encoded SNPs where NetDiseaseSNP and SIFT agree on the prediction; (2) All Blosum62 data encoded SNPs where NetDiseaseSNP and SIFT disagree on the prediction; (3) All Blosum62 data encoded SNPs where SIFT can generate a prediction. Table S1da Performance of NetDiseaseSNP. (1) All Blosum62 data encoded SNPs where SIFT predictions exist and NetDiseaseSNP and SIFT agree on the prediction; (2) All Blosum62 data encoded SNPs where SIFT predictions exist and NetDiseaseSNP and SIFT disagree on the prediction; (3) All Blosum62 data encoded SNPs where SIFT predictions [file pone.0068370.s001.doc]

# Supplementary information

## Performance of NetDiseaseSNP and SIFT on running evaluation dataset

NetDiseaseSNP relies on the calculations from SIFT – it is therefore necessary and highly relevant to compare the performances of the two predictors. The performance of our predictor was measured on the running evaluation set. The overall performances of the two predictors are not directly comparable since Blosum62 matrix data has been used as input to NetDiseaseSNP for SNPs where SIFT was not able to generate output or the protein was longer than 2000 amino acids. In order to compare the two predictors it is therefore necessary to split the performances of both predictors based on the encoding of the SNPs used for NetDiseaseSNP. Such a performance splitting is shown in the tables below.

Each of the first 4 subsections below contain two tables with performance data for either NetDiseaseSNP or SIFT on different groupings of polymorphisms based on the encoding of polymorphisms used in the input to our predictor. The encoding of the SNPs mentioned in the caption of tables thus refers to the encoding used by NetDiseaseSNP. The tables in each subsection further split the performance data depending on the encoding of polymorphisms used by the given prediction method (first column) and the combination of predictions made by the two predictors (second column).

The heading for each subsection describes the group of SNPs which are split in the given subsection and the performance of NetDiseaseSNP on all SNPs in this group category is given in the last line in the table for NetDiseaseSNP. The other lines in the table for our predictor and all lines in the table for SIFT in the given subsection are subsets of the quantity of SNP mentioned in the subsection headline.

Note that the first two lines in the tables for both predictors are directly comparable since it is exactly the same variants that are predicted on by the two predictors. This is not the case for the other lines in the two tables in the subsections due to SIFT not being able to generate predictions for all variants. NetDiseaseSNP is able to generate a prediction for all variants from all proteins due to the use of Blosum62 matrix data for variants where SIFT output is not available furthermore Blosum62 matrix data is also used by our predictor to encode variants from proteins longer than 2000 amino acids.

Table S1e summarizes the number of neutral and disease SNPs for each of the different types of encoding for SNPs.

### Performance on all SNPs – both SIFT and Blosum62 data encoded SNPs

The last row in Table S1aa shows the performance data for NetDiseaseSNP on all SNPs in the dataset – both SIFT and Blosum62 matrix data encoded SNPs. This line thus shows the overall performance of our predictor on the running evaluation set. The last line in Table S1ab shows the overall performance of SIFT on all SNPs in the evaluation set where SIFT is able to generate a prediction.

| **Input data:** | **Agree** | **TP** | **FP** | **TN** | **FN** | **MCC** | **SEN** | **SPE** |
| --- | --- | --- | --- | --- | --- | --- | --- | --- |
| SIFT/Blosum (1) | Yes | 27855 | 4339 | 30270 | 5726 | 0.705 | 0.830 | 0.875 |
| SIFT/Blosum (2) | No | 2242 | 1977 | 1589 | 1649 | 0.022 | 0.576 | 0.446 |
| Blosum (3) | ----- | 961 | 155 | 340 | 237 | 0.469 | 0.802 | 0.687 |
| SIFT/Blosum (4) | ----- | 31058 | 6471 | 32199 | 7612 | 0.636 | 0.803 | 0.833 |

**Table S1aa**: Performance of NetDiseaseSNP. (1) All SNPs where NetDiseaseSNP and SIFT agree on the prediction; (2) All SNPs where NetDiseaseSNP and SIFT disagree on the prediction; (3) All SNPs where SIFT is not able to generate a prediction; (4) All SNPs.

| **Input data:** | **Agree** | **TP** | **FP** | **TN** | **FN** | **MCC** | **SEN** | **SPE** |
| --- | --- | --- | --- | --- | --- | --- | --- | --- |
| SIFT (1) | Yes | 27855 | 4339 | 30270 | 5726 | 0.705 | 0.830 | 0.875 |
| SIFT (2) | No | 1649 | 1589 | 1977 | 2242 | -0.022 | 0.424 | 0.554 |
| SIFT (3) | ----- | 29504 | 5928 | 32247 | 7968 | 0.633 | 0.787 | 0.845 |

**Table S1ab**: Performance of SIFT. (1) All SNPs where NetDiseaseSNP and SIFT agree on the prediction; (2) All SNPs where NetDiseaseSNP and SIFT disagree on the prediction; (3) All SNPs where SIFT can generate a prediction.

### Performance on SIFT data encoded SNPs

| **Input data:** | **Agree** | **TP** | **FP** | **TN** | **FN** | **MCC** | **SEN** | **SPE** |
| --- | --- | --- | --- | --- | --- | --- | --- | --- |
| SIFT (1) | Yes | 25446 | 4105 | 27468 | 5141 | 0.703 | 0.832 | 0.870 |
| SIFT (2) | No | 1783 | 1052 | 1326 | 798 | 0.251 | 0.691 | 0.558 |
| SIFT (3) | ----- | 27229 | 5157 | 28794 | 5939 | 0.669 | 0.821 | 0.848 |

**Table S1ba**: Performance of NetDiseaseSNP. (1) All SIFT data encoded SNPs where NetDiseaseSNP and SIFT agree on the prediction; (2) All SIFT data encoded SNPs where NetDiseaseSNP and SIFT disagree on the prediction; (3) All SIFT data encoded SNPs.

| **Input data:** | **Agree** | **TP** | **FP** | **TN** | **FN** | **MCC** | **SEN** | **SPE** |
| --- | --- | --- | --- | --- | --- | --- | --- | --- |
| SIFT (1) | Yes | 25446 | 4105 | 27468 | 5141 | 0.703 | 0.832 | 0.870 |
| SIFT (2) | No | 798 | 1326 | 1052 | 1783 | -0.251 | 0.309 | 0.442 |
| SIFT (3) | ----- | 26244 | 5431 | 28520 | 6924 | 0.632 | 0.791 | 0.840 |

**Table S1bb**: Performance of SIFT. (1) All SIFT data encoded SNPs where NetDiseaseSNP and SIFT agree on the prediction; (2) All SIFT data encoded SNPs where NetDiseaseSNP and SIFT disagree on the prediction; (3) All SIFT data encoded SNPs where SIFT can generate a prediction.

### Performance on Blosum62 data encoded SNPs

| **Input data:** | **Agree** | **TP** | **FP** | **TN** | **FN** | **MCC** | **SEN** | **SPE** |
| --- | --- | --- | --- | --- | --- | --- | --- | --- |
| Blosum (1) | Yes | 2409 | 234 | 2802 | 585 | 0.733 | 0.805 | 0.923 |
| Blosum (2) | No | 459 | 925 | 263 | 851 | -0.430 | 0.350 | 0.221 |
| Blosum (3) | ----- | 961 | 155 | 340 | 237 | 0.469 | 0.802 | 0.687 |
| Blosum (4) | ----- | 3829 | 1314 | 3405 | 1673 | 0.416 | 0.696 | 0.722 |

**Table S1ca**: Performance of NetDiseaseSNP. (1) All Blosum62 data encoded SNPs where NetDiseaseSNP and SIFT agree on the prediction; (2) All Blosum62 data encoded SNPs where NetDiseaseSNP and SIFT disagree on the prediction; (3) All Blosum62 data encoded SNPs where SIFT is not able to generate a prediction; (4) All Blosum62 data encoded SNPs.

| **Input data:** | **Agree** | **TP** | **FP** | **TN** | **FN** | **MCC** | **SEN** | **SPE** |
| --- | --- | --- | --- | --- | --- | --- | --- | --- |
| SIFT (1) | Yes | 2409 | 234 | 2802 | 585 | 0.733 | 0.805 | 0.923 |
| SIFT (2) | No | 851 | 263 | 925 | 459 | 0.430 | 0.650 | 0.779 |
| SIFT (3) | ----- | 3260 | 497 | 3727 | 1044 | 0.644 | 0.757 | 0.882 |

**Table S1cb**: Performance of SIFT. (1) All Blosum62 data encoded SNPs where NetDiseaseSNP and SIFT agree on the prediction; (2) All Blosum62 data encoded SNPs where NetDiseaseSNP and SIFT disagree on the prediction; (3) All Blosum62 data encoded SNPs where SIFT can generate a prediction.

### Performance on Blosum62 data encoded SNPs where SIFT predictions exist

Notice that the number of SNPs in this subsection is a subset of the number of SNPs in the previous section. The reason for SIFT output not being used as input to our predictor for these SNPs even though SIFT output does exists, is that the SNPs originate from proteins longer than 2000 amino acids.

| **Input data:** | **Agree** | **TP** | **FP** | **TN** | **FN** | **MCC** | **SEN** | **SPE** |
| --- | --- | --- | --- | --- | --- | --- | --- | --- |
| Blosum (1) | Yes | 2409 | 234 | 2802 | 585 | 0.733 | 0.805 | 0.923 |
| Blosum (2) | No | 459 | 925 | 263 | 851 | -0.430 | 0.350 | 0.221 |
| Blosum (3) | ----- | 2868 | 1159 | 3065 | 1436 | 0.393 | 0.666 | 0.726 |

**Table S1da**: Performance of NetDiseaseSNP. (1) All Blosum62 data encoded SNPs where SIFT predictions exist and NetDiseaseSNP and SIFT agree on the prediction; (2) All Blosum62 data encoded SNPs where SIFT predictions exist and NetDiseaseSNP and SIFT disagree on the prediction; (3) All Blosum62 data encoded SNPs where SIFT predictions exist.

| **Input data:** | **Agree** | **TP** | **FP** | **TN** | **FN** | **MCC** | **SEN** | **SPE** |
| --- | --- | --- | --- | --- | --- | --- | --- | --- |
| SIFT (1) | Yes | 2409 | 234 | 2802 | 585 | 0.733 | 0.805 | 0.923 |
| SIFT (2) | No | 851 | 263 | 925 | 459 | 0.430 | 0.650 | 0.779 |
| SIFT (3) | ----- | 3260 | 497 | 3727 | 1044 | 0.644 | 0.757 | 0.882 |

**Table S1db**: Performance of SIFT. (1) All Blosum62 data encoded SNPs where SIFT predictions exist and NetDiseaseSNP and SIFT agree on the prediction; (2) All Blosum62 data encoded SNPs where SIFT predictions exist and NetDiseaseSNP and SIFT disagree on the prediction; (3) All Blosum62 data encoded SNPs where SIFT predictions exist and SIFT can generate a prediction.

### SNP count for different types of encoding for SNPs

Table S1e shows the number of known neutral and disease SNPs in the evaluation set for each of the different types of encoding used by NetDiseaseSNP.

| **Input data:** | **Long Protein** | **All SNPs SIFT data** | **Neutral**  **SNPs** | **Disease**  **SNPs** | **Total**  **SNPs** |
| --- | --- | --- | --- | --- | --- |
| SIFT/Blosum (1) | ----- | No | 38670 | 38670 | 77340 |
| SIFT (2) | No | Yes | 33951 | 33168 | 67119 |
| Blosum (3) | ----- | No | 4719 | 5502 | 10221 |
| Blosum (4) | Yes | Yes | 4224 | 4304 | 8528 |
| Blosum (5) | ----- | No | 495 | 1198 | 1693 |

**Table S1e**: Number of neutral and disease SNPs for each of different types of encoding for SNPs. The columns in the table are: Column 1: Input data to NetDiseaseSNP; Column 2: Protein is longer than 2000 amino acids; Column 3: SIFT data exists for the all SNPs; Column 4: Number of neutral SNPs; Column 5: Number of disease SNPs. The rows in the table are: (1) All SNPs – both SIFT and Blosum62 data encoded SNPs; (2) SIFT data encoded SNPs; (3) Blosum62 data encoded SNPs; (4) Blosum62 data encoded SNPs where SIFT output data exists i.e. protein is longer than 2000 amino acids; (5) Blosum62 data encoded SNPs where SIFT output data does not exist.

### Discussion of performance on running evaluation set

When viewing the tables above it is important to be aware that all SNPs in all proteins also SNPs in proteins longer than 2000 amino acids, are submitted to the SIFT alignment step. For some of these SNPs, SIFT will not be able to generate predictions. NetDiseaseSNP on the other hand can make predictions for all polymorphisms including those where SIFT output is not available and in these cases we encode the polymorphisms using a Blosum62 matrix. Additionally, variations from proteins longer than 2000 amino acids are encoded with Blosum62 matrix data, independently of the existence of SIFT predictions. The use of Blosum62 matrix data makes it complicated to compare the performances of the two predictors but it ensures that NetDiseaseSNP always is able to give a prediction.

An overall performance comparison shows that the use of Blosum62 matrix data to encode variants causes the overall performance of our predictor, MCC=0.64, to drop down to the level of SIFT, MCC=0.63, as can be seen from Table S1aa and Table S1ab. Notice though from Table S1ca that NetDiseaseSNP has some prediction potential for Blosum62 data encoded variants but since the performance is MCC=0.42, the user should use the predictions from our predictor for Blosum62 data encoded variants with some caution.

Notice also from all tables (Table S1aa to Table S1da) that the performance for NetDiseaseSNP is close to MCC=0.70 for all types of SNP encoding when both predictors agree on a prediction which is considerable higher than the overall performance for NetDiseaseSNP as well as for SIFT.

In both Table S1aa and Table S1ba the performance of NetDiseaseSNP is higher than the performance of SIFT when the two predictors disagree on the prediction – in fact SIFT is most often wrong in these cases. However the use of Blosum62 matrix data to encode some of the SNPs in this category of SNPs in Table S1aa causes this difference in performance between NetDiseaseSNP and SIFT to almost disappear. Notice that this is not the case in Table S1ba where Blosum62 encoded SNPs are not included. It can hence be concluded that when alignment data gathered by SIFT is used as input to our predictor, a higher predictive performance is achieved for our predictor than for SIFT.

As can be seen from Table S1ca and Table S1da the performance of NetDiseaseSNP with an MCC=0.70 is exceeded for polymorphisms where both predictors agree on the prediction and Blosum62 matrix data has been used as input to NetDiseaseSNP even though SIFT is able to generate predictions (polymorphisms in proteins longer than 2000 amino acids). However for this type of polymorphisms our predictor has a lower performance than SIFT when the two predictors disagree on the prediction – in fact NetDiseaseSNP is most often wrong. This result is not unexpected since the study by Ng & Henikoff (2001) showed that the performance of disease SNP prediction on Blosum62 matrix data is low compared to prediction based on sequence specific information such as the alignment created by SIFT. This is also evident in Table S1da and Table S1db where the performances for both predictors are shown for polymorphisms where Blosum62 encoding has been used for NetDiseaseSNP even though SIFT was able to generate a prediction. For these types of polymorphisms the performance of our predictor shown in Table S1da is thus MCC=0.39 while the performance of SIFT shown in Table S1db is MCC=0.64.

The user should be aware that in the current implementation of NetDiseaseSNP, SIFT output will not be considered for variations in proteins longer than 2000 amino acids even though SIFT might in fact be able to generate predictions for some of these variations. SNPs in proteins longer than 2000 amino acids are encoded with Blosum62 matrix data in the input to NetDiseaseSNP in order to decrease the CPU-time. Furthermore it was also observed that SIFT was unable to score these SNPs more often than SNPs from proteins shorter than 2000 amino acids. When proteins longer than 2000 amino acids were cut into smaller parts (without considering division into e.g. protein domains) with overlapping regions between the parts, then the predictions by SIFT in the overlapping regions were often found to be inconsistent. The reason for the lower performance of SIFT on proteins longer than 2000 amino acids was too few sequences in the alignment for some positions in the query protein. The obvious explanation for SIFT being unable to find enough related sequences for certain regions of long proteins is that there is in fact too few related sequences for these regions in long proteins. Notice though that most proteins in the human proteome are shorter than 2000 amino acids so the use of Blosum62 matrix data encoding of variants due to long proteins will not be an issue for most variants.

Hence Table S1e shows that NetDiseaseSNP uses SIFT output data encoding for 67119 SNPs out 77340 SNPs and only uses Blosum62 matrix data encoding for 10221 SNPs out of 77340 SNPs on the running evaluation set. From Table S1ba and Table S1bb it can be seen that NetDiseaseSNP most often will generate predictions based on SIFT matrix data where NetDiseaseSNP and SIFT agree on the prediction. The performance of NetDiseaseSNP in terms of MCC on this group of variants is 0.70 as shown in Table S1ba.

Table S1e also shows that 8528 polymorphisms out of 10221 Blosum62 encoded polymorphisms occurs in proteins longer than 2000 amino acids where SIFT is able to generate predictions for these polymorphisms. It is thus only 1693 variations (in both long and short proteins) out of 77340 variations where SIFT is unable to generate a prediction. The strongest argument for using Blosum62 encoding for variations from proteins longer than 2000 amino acids is thus to reduce the CPU-time used by NetDiseaseSNP. However as mentioned above the performance of SIFT can be low for variations in long proteins if these variations occur in regions with too few related sequences in the alignment.

## Prediction of cancer driver mutations in the COSMIC dataset

The ability of our method to distinguish cancer driver and passenger mutations was initially tested on version v44 (November 2009) of the COSMIC cancer mutation dataset (Forbes *et al.,* 2008). We assume that cancer driver mutations are predicted as disease polymorphisms and that passenger mutations are predicted as neutral polymorphisms.

Table S2a shows results for all possible combinations of predictions by NetDiseaseSNP and SIFT on the COSMIC dataset and lists the number of variations in each category. Notice though NetDiseaseSNP does not run SIFT on proteins longer than 2000 amino acids. The tables below therefore do not show predictions by SIFT on variations from such proteins even though SIFT might be able to generate predictions for some variations in some of these proteins.

Table S2b and Table S2c summarize protein characteristics concerning Blosum62 matrix encoded variants. For each type of protein category the number of variants predicted to be disease (Table S2b) and neutral (Table S2c) variants are shown.

| **Description** | **NetDiseaseSNP** | **SIFT** | **Mutations** |
| --- | --- | --- | --- |
| (1) | Disease | Disease | 3345 |
| (2) | Neutral | Neutral | 1886 |
| (3) | Disease | Neutral | 357 |
| (4) | Neutral | Disease | 305 |
| (5) | Disease | -------- | 372 |
| (6) | Neutral | -------- | 462 |
| (7) Total | -------- | -------- | 6727 |

**Table S2a**: Predictions by NetDiseaseSNP and SIFT. The rows in the table are: (1) Both NetDiseaseSNP and SIFT predict disease; (2) Both NetDiseaseSNP and SIFT predict neutral; (3) NetDiseaseSNP predicts disease and SIFT predicts neutral; (4) NetDiseaseSNP predicts neutral and SIFT predicts disease; (5) NetDiseaseSNP predicts disease and SIFT no prediction; (6) NetDiseaseSNP predicts neutral and SIFT no prediction; (7) Total number of mutations.

| **Description** | **Long Protein** | **Blosum Protein** | **Mutations** |
| --- | --- | --- | --- |
| (1) | Yes | Yes | 357 |
| (2) | No | Yes | 13 |
| (3) | No | No | 2 |
| (4) Total | -------- | -------- | 372 |

**Table S2b**: Predictions by NetDiseaseSNP on mutations where NetDiseaseSNP predicts the mutation to be a disease mutation and mutations are encoded with Blosum62 matrix data. The columns in the table are: Column 1: Description of the data in the row; Column 2: Protein is longer than 2000 amino acids; Column 3: All mutations at all positions in the protein are encoded with Blosum62 matrix data. The rows in the table are: (1) Protein longer than 2000 amino acids; (2) SIFT is not able to generate output for any mutation in this protein and the protein is shorter than 2000 amino acids; (3) SIFT is able to generate output for some mutations in this protein and the protein is shorter than 2000 amino acids; (4) Total number of mutations.

| **Description** | **Long Protein** | **Blosum Protein** | **Mutations** |
| --- | --- | --- | --- |
| (1) | Yes | Yes | 447 |
| (2) | No | Yes | 11 |
| (3) | No | No | 4 |
| (4) Total | -------- | -------- | 462 |

**Table S2c**: Predictions by NetDiseaseSNP on mutations where NetDiseaseSNP predicts the mutation to be a neutral mutation and mutations are encoded with Blosum62 matrix data. The columns in the table are: Column 1: Description of the data in the row; Column 2: Protein is longer than 2000 amino acids; Column 3: All mutations at all positions in the protein are encoded with Blosum62 matrix data. The rows in the table are: (1) Protein longer than 2000 amino acids; (2) SIFT is not able to generate output for any mutation in this protein and the protein is shorter than 2000 amino acids; (3) SIFT is able to generate output for some mutations in this protein and the protein is shorter than 2000 amino acids; (4) Total number of mutations.

Table S2a shows that NetDiseaseSNP in agreement with SIFT predicts 3345 out of 6727 cancer mutations from the COSMIC dataset to be disease causing. However both predictors agree on predicting 1886 of the mutations not be disease causing. These predictions are as shown in Table S1ba made with a performance of MCC=0.70 for the running evaluation set. The COSMIC dataset can be assumed to be enriched for driver mutations as compared to large scale somatic mutation discovery datasets which can be expected to contain a fair number of passenger mutations (Lee *et al.,* 2009). It is therefore on the other hand reasonable to suggest that taken the high performance of NetDiseaseSNP on such variations into account then at least some of these variations which are predicted to be neutral variations could in fact be passenger mutations – since the COSMIC cancer dataset might contain passenger mutations which have not been filtered out.

Overall NetDiseaseSNP thus predicts including Blosum62 encoded mutations, 4074 mutations to be disease causing and 2653 mutations not to be disease causing in the COSMIC dataset. A two-sided binomial test for variants encoded with SIFT output as well as Blosum62 matrix data is performed where a prediction by NetDiseaseSNP of a given mutation to be disease causing is counted as a success and a prediction by NetDiseaseSNP of a given mutation not to be disease causing is counted as a failure. The p-value for NetDiseaseSNP being able to predict cancer variants on these criteria is: p-value < 2.2∙10^-16^. It is therefore fair to conclude that NetDiseaseSNP can predict cancer driver mutations with a performance significantly above random – especially when it is taken into account that some of the predicted neutral variants might in fact be passenger mutations and should thus not be counted as failures but instead as successes in the two-sided binomial test.

Table S2a also shows that for mutations where both predictors disagree on the prediction (and SIFT output has been used as input to NetDiseaseSNP), our predictor predicts 357 mutations to be disease causing while SIFT predicts 305 mutations to be disease causing. A two sided binomial test is performed where a prediction by NetDiseaseSNP of a given mutation to be disease causing is counted as a success and a prediction by our predictor of a given mutation not to be disease causing is counted as a failure. The p-value=0.047 for this test shows that NetDiseaseSNP predicts significantly more disease causing polymorphisms than neutral polymorphisms for variants where the two predictors disagree. The p-value also shows that NetDiseaseSNP predicts significantly more disease causing variations than SIFT on this group of variations – in fact SIFT is shown to predict significantly more neutral variants than disease causing variants. The COSMIC dataset can as mentioned above, be assumed to be enriched for driver mutations as compared to large scale somatic mutation discovery datasets (Lee *et al.,* 2009). The result of the two-sided binomial test therefore suggests that NetDiseaseSNP is better than SIFT at predicting cancer driver mutations since NetDiseaseSNP recognizes more of these cancer driver mutations as disease polymorphisms than SIFT.

For mutations encoded with Blosum62 matrix data our predictor predicts 372 disease and 462 neutral mutations (see Table S2a) indicating that predictions based on Blosum62 matrix data should be used with caution.

Notice from Table S2b and Table S2c that most of these Blosum62 encoded mutations come from proteins which are longer than 2000 amino acids. Only 15 Blosum62 encoded variants predicted to be disease variants and 15 Blosum62 encoded variants predicted to be neutral variants occur in proteins shorter than 2000 amino acids. This indicates that SIFT most often will be able to generate a prediction for variants in proteins shorter than 2000 amino acids which also comprise most proteins in the human proteome.

## Performance of NetDiseaseSNP and SIFT on dataset from CanPredict

The ability of NetDiseaseSNP and SIFT to distinguish cancer driver mutations from passenger mutations was investigated in more detail on a dataset developed for the creation of the cancer-associated missence mutation predictor called CanPredict (Kaminker *et al.,* 2007). This dataset consists of cancer mutations from the COSMIC cancer dataset (Forbes *et al.,* 2006) which are assumed to be driver mutations and SNPs from dbSNP (Sherry *et al.,* 2001) which are assumed to be passenger mutations.

Table S3a and Table S3b below contain performance data for the two predictors on the CanPredict dataset. These two tables split the performance data depending on the encoding of the polymorphisms used by the given prediction method and the combination of predictions made by NetDiseaseSNP and SIFT. The last line in Table S3a shows the performance of our predictor on all polymorphisms in the CanPredict dataset whereas the other lines in the table for NetDiseaseSNP, Table S3a, and all lines in the table for SIFT, Table S3b, are subsets of this quantity of polymorphisms. Notice that the first two lines in the tables for the two predictors are directly comparable since it is exactly the same variations that are predicted on by both predictors. This is not the case for the other lines in the two tables due to SIFT not being able to generate predictions for all variations and SIFT not being run on variations from proteins longer than 2000 amino acids. NetDiseaseSNP is able to generate a prediction for all variations from all proteins due to the use of Blosum62 matrix data for variations where SIFT output is not available.

| **Input data:** | **Agree** | **TP** | **FP** | **TN** | **FN** | **MCC** | **SEN** | **SPE** |
| --- | --- | --- | --- | --- | --- | --- | --- | --- |
| SIFT (1) | Yes | 742 | 298 | 2531 | 151 | 0.691 | 0.831 | 0.895 |
| SIFT (2) | No | 53 | 87 | 149 | 25 | 0.270 | 0.680 | 0.631 |
| Blosum (3) | ----- | 13 | 103 | 236 | 13 | 0.108 | 0.500 | 0.696 |
| SIFT/Blosum (4) | ----- | 808 | 488 | 2916 | 189 | 0.613 | 0.810 | 0.857 |

**Table S3a**: Performance of NetDiseaseSNP. (1) All mutations where NetDiseaseSNP and SIFT agree on the prediction; (2) All mutations where NetDiseaseSNP and SIFT disagree on the prediction; (3) All mutations where SIFT is not able to generate a prediction; (4) All mutations.

| **Input data:** | **Agree** | **TP** | **FP** | **TN** | **FN** | **MCC** | **SEN** | **SPE** |
| --- | --- | --- | --- | --- | --- | --- | --- | --- |
| SIFT (1) | Yes | 742 | 298 | 2531 | 151 | 0.691 | 0.831 | 0.895 |
| SIFT (2) | No | 25 | 149 | 87 | 53 | -0.270 | 0.321 | 0.369 |
| SIFT (3) | ----- | 767 | 447 | 2618 | 204 | 0.600 | 0.790 | 0.854 |

**Table S3b**: Performance of SIFT. (1) All mutations where NetDiseaseSNP and SIFT agree on the prediction; (2) All mutations where NetDiseaseSNP and SIFT disagree on the prediction; (3) All mutations where SIFT can generate a prediction.

The performance of NetDiseaseSNP of MCC=0.70 in Table S1ba on variants where both predictors agree on the prediction and SIFT output has been used as input to NetDiseaseSNP is almost retained on cancer driver and passenger mutations with MCC=0.69 as shown in Table S3a. The same can be said for the performance of our predictor on the variants where both predictors disagree on the prediction and SIFT output has been used as input to our predictor (compare Table S3a and Table S1ba).

NetDiseaseSNP has a slightly higher overall performance on the CanPredict dataset in terms MCC of 0.61 than SIFT which has an overall performance of MCC=0.60 (see Table S3a and Table S3b). It is thus fair to conclude that both predictors have the ability to distinguish cancer driver mutations from passenger mutations. The difference in overall performance between the two predictors is not large however it should be noted that the performance of NetDiseaseSNP also contains predictions for mutations where no SIFT prediction is available and therefore Blosum62 matrix data is used as input to NetDiseaseSNP. Performance of disease mutation prediction on Blosum62 matrix data has previously been shown to be low compared to sequence specific information such as the alignment created by SIFT (Ng & Henikoff, 2001) which is also found to be the case for our predictor for this dataset: MCC=0.11 (see Table S3a). The overall performance of NetDiseaseSNP of MCC=0.61 is thus lowered by these Blosum62 matrix data encoded polymorphisms compared to the overall performance for SIFT of MCC=0.60 since these Blosum62 matrix data encoded polymorphisms are not included in the performance for SIFT. SIFT is only run on proteins shorter than 2000 amino acids for the CanPredict dataset so there is no need for splitting the performance any further as was the case for the evaluation set performance shown in Table S1aa to Table S1db.

The difference in performance between the two predictors is highlighted by the fact that the MCC for polymorphisms where both predictors disagree is negative for SIFT (MCC=-0.27) and positive for NetDiseaseSNP (MCC=+0.27). To test whether our predictor is better than SIFT at distinguishing driver mutations from passenger mutations a two-sided binomial test is performed for the polymorphisms where both predictors disagree and SIFT output has been used as input to NetDiseaseSNP. A prediction for a mutation where our predictor generates the correct prediction is counted as a success (202 mutations) and a mutation where SIFT generates the correct prediction is counted as a failure (112 mutations). The p-value for NetDiseaseSNP being better than SIFT at distinguishing cancer driver mutations from passenger mutations is: p-value=4∙10^-7^. This result shows that our predictor is significantly better than SIFT at distinguishing cancer driver mutations from passenger mutations.

In conclusion the results in Table S3a and Table S3b therefore show that NetDiseaseSNP as well as SIFT can distinguish cancer driver mutations from passenger mutations and also suggest that NetDiseaseSNP is better at distinguishing cancer driver mutations from passenger mutations than SIFT. The reason for this higher performance is most likely due to our predictor using at least some of its potential to extract all relevant information in the alignment created by SIFT – and SIFT not having this potential since SIFT only uses the normalized probability for the mutated amino acid to generate the prediction.

## References

Forbes,S., Clements,J., Dawson,E., Bamford,S., Webb,T., Dogan,A., Flanagan,A., Teague,J., Wooster,R., Futreal,P.A. and Stratton,M.R. (2006) COSMIC 2005. *Br J Cancer*, **94**, 318–322.

Forbes,S.A., Bhamra,G., Bamford,S., Dawson,E., Kok,C., Clements,J., Menzies,A., Teague,J.W., Futreal,P.A. and Stratton,M.R. (2008) The Catalogue of Somatic Mutations in Cancer (COSMIC). *Curr Protoc Hum Genet*, **Chapter 10**, Unit 10.11.

Kaminker,J.S., Zhang,Y., Watanabe,C. and Zhang,Z. (2007) CanPredict: a computational tool for predicting cancer-associated missense mutations. *Nucleic Acids Res*, **35**, W595–598.

Lee,W., Zhang,Y., Mukhyala,K., Lazarus,R.A. and Zhang,Z. (2009) Bidirectional SIFT predicts a subset of activating mutations. *PLoS ONE*, **4**, e8311.

Ng,P.C. & Henikoff,S. (2001) Predicting deleterious amino acid substitutions. *Genome Res*, **11**, 863–874.

Sherry,S.T., Ward,M.H., Kholodov,M., Baker,J., Phan,L., Smigielski,E.M. and Sirotkin,K. (2001) dbSNP: the NCBI database of genetic variation. *Nucleic Acids Res*, **29**, 308–311.
